# Effects of Compound Sand Barrier for Habitat Restoration on Sediment Grain-size Distribution in Ulan Buh Desert

**DOI:** 10.1038/s41598-020-59538-7

**Published:** 2020-02-13

**Authors:** Xia Pan, Zhenyi Wang, Yong Gao

**Affiliations:** 1 College of Desert Control Science and Engineering, Inner Mongolia Agricultural University, Hohhot, 010018 China; 2 Wind Erosion Key Laboratory of Central and Government, Hohhot, 010018 China

**Keywords:** Forestry, Environmental impact

## Abstract

Wind erosion is a huge challenge for ecologists to stabilize sand dunes and to change them into stable productive ecosystems. In order to better understand its role in the process of ecological restoration, the sediment grain-size characteristics of compound sand barrier were evaluated through field experimental observation. The results indicated that the compound sand barrier was mainly composed of extremely fine sand and fine sand, and the fine sand and extremely fine sand in the inner side were higher than the east and west sides of the compound sand barrier. Due to the blocking effect of compound sand barrier, the Sorting Coefficient became better, the Skewness belonged to the positive deviation and the Kurtosis presented leptokurtosis distribution. Moreover, while the cumulative frequency distribution curve in the inner side became steeper, the slope increased and reached the top of the curve ahead of time. The effect of wind environment and vegetation coverage on the surface sediments showed that the average annual wind velocity and vegetation coverage was negatively correlated with the average grain-size, but positively correlated with the Sorting Coefficient. There was a significant correlation among the annual wind speed, vegetation coverage, average grain-size and Sorting Coefficient, which indicated that vegetation coverage and wind environment was the key factor leading to the difference of surface sediments in this area. Collectively, the establishment of compound sand barrier is one of the most effective methods of sand-fixing with engineering measure in the arid desert regions. Therefore, given the complexities of agricultural systems, stubble retention and black film covered during harvesting and incorporation of the stubble into soil in the next spring appears to be the best choice in the dry northern China where farmlands suffer serious wind erosion.

## Introduction

China is one of the countries most affected by desertification. Since the early 1950s, more than 70 serious sandstorms have occurred, which have caused huge losses to human life and property, and seriously threatened the living environment of the local people. Sandy desert land cover a 1.49 million km^2^ of China, 15.5% of the total area^1,2^. Due to sand transport and dune burial on arid desert regions, pipeline corridors, power transmission lines, transportation routes and people exploitation have been damaged. So far, wind erosion is still one of the most serious problems in many agricultural lands^3,4^ and it is currently recognized as a major source of environment degradation^5^. Consequently, a series of ecological engineering constructions and projects to stabilize sand dunes along highways or railways in desert regions were begun in the 1950s^6,7^. The establishment of sand barriers on deserted land has been one of the most popular restoration techniques. It has been widely used in habitat recovery and protection before and after the construction, such as pipeline corridors, transmission lines and transport routes in arid desert areas, as well as post-mining landscapes^8,9^. Its main function is to reduce wind speed and achieve the purpose of wind prevention and sand fixation by changing the properties of underlying surface and increasing surface roughness^3^.

More specifically, sand barriers with grass grid are a widely used engineering measure with sand control. On the one hand, it can effectively control the movement of sand particles by increasing the surface aerodynamic roughness and decreasing the surface wind speed to below the sand speed. On the other hand, by blocking the wind-sand flow and reducing the sand carrying capacity of the wind, the movement of wind-sand can be controlled. At the same time, the settlement of fine particles also makes the soil particles in the barrier refine, which is beneficial to the restoration of vegetation^10^. In contrast, PLA (Poly Lactic Acid) is a kind of environmentally friendly and sand fixation material with wide application prospect. After the sand flow enters into the barrier grid of PLA, the kinetic energy of the fluid is greatly weakened and a low-speed settling zone is formed behind the barrier, and the particles settle near the barrier. In the meantime, vortex current is formed in the barrier, which promotes the development of stable erosion datum in the barrier^11^. Further, chemical sand fixation can rapidly form a consolidation layer with wind erosion prevention on the surface of quicksand and promote plant growth by improving the hydro-thermal conditions of sandy land, enhancing the biological activity and aggregate structure of sandy land, avoiding the leakage of water and fertilizer and preventing the upward migration of deep salt^12^. As a whole, plant sand fixation is the most widely used measure, which has the characteristics of lasting, stable and effective^13^. In China, tillage has long been an important agricultural activity for preparing seed beds, mixing fertilizers and crop residues into the soil, alleviating soil compaction and controlling the spread of weeds^14–16^. Some tillage measures can reduce wind erosion rates by producing soil blocks and aggregates that decrease wind erosion rate^17–19^. Many studies have shown that no tillage with stubble retention can reduce wind erosion by decreasing the wind drag on the soil surface, intercepting moving soil particles from the surface and physically protecting the surface from blown-in sand^20^.

Soil wind erosion processes include mechanical process and dynamic changes of the factors affecting soil wind erosion, as well as the corresponding changes of wind erosion rate^21^. Wind erosion involved the processes of detachment, transport and deposition under the effects of wind velocity^8^. One key parameter affecting wind erosion rate is sediment particle size^22^. The composition of particles in different diameter grades of surface sediments is the key factor affecting surface wind erosion activity, and it plays an important role in the underlying surface factors of surface wind erosion process and is also an indicator of the occurrence and development of land desertification^23–27^. The spatial difference of sediment particle size parameters directly reflects the transport and accumulation process experienced by sediments, and even has a certain indication significance for the source of sand dunes^28^. The parameters variation is controlled by transport medium, transportation mode, sedimentary environment and climate^29^. Moreover, the sediment particle size eroded from arid and semi-arid areas provides basic information about erosion processes, and the measures to control the surface wind and sediment transport can be put forward^30,31^. As an important physical property of surface particles, the proportion and distribution of sediment particle size in different diameter levels are more and more widely used in the research of land desertification^32^.

The Ulan Buh Desert is located in the upper reaches of the Yellow River in Inner Mongolia Autonomous Region and develops a sandy riverbed^33^. This area is drought and less rain in spring, strong wind and sand activity, deep dry soil layer before sowing and low soil water content. The water requirement for emergence is misplaced, which often results in incomplete seedling emergence and seriously affects the yield and quality of corn. Plastic film annual mulching of the ground can not only reduce the ineffective dissipation of water in winter and spring, provide water protection for corn sowing in suitable time, but also reduce wind speed and prevent sand on the surface^19^. The compound sand barrier used in this study is the combination of straw and plastic film. The variation characteristics of surface sediment grain size with compound sand barriers in Ulan Buh Desert were discussed from the aspects of material and laying mode, which could not only find out the change law of wind and sand flow after being hindered by wind sand flow from the mechanism point of view, but also helpful to clarify the spatial distribution of wind and sediment activities and accurately evaluate of protection benefit with compound sand barrier. It provides theoretical support for the large-scale popularization and application of compound sand barrier in the future.

## Site Description

**Figure 1:**
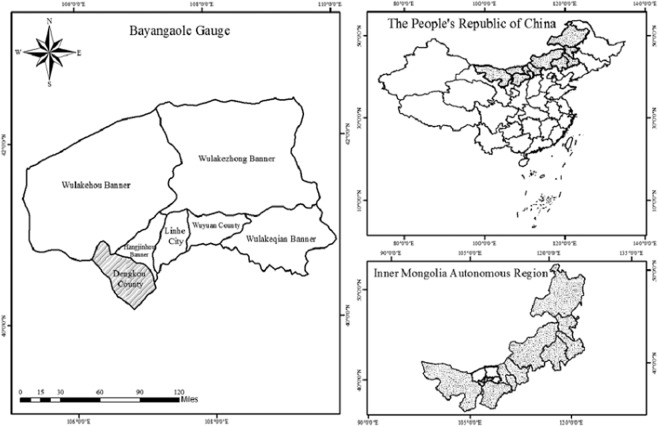
Field survey and sampling location map of the study area.

**Figure 2:**
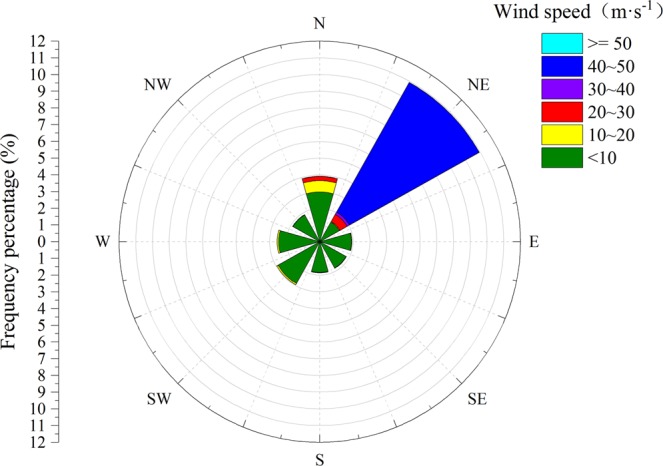
Wind rose at northeastern margin region of Ulan Buh Desert from 2011 to 2018.

This study area is located in Dengkou County in the Bayangaole gauge region at the northeast edge of the Ulan Buh Desert (40°26′30″N, 106°25′9″E) (Fig. 1). It has a typical continental arid climate. The annual precipitation is less than 100 mm (mostly concentrated between July and August) and the aridity index is 6.3^34^. According to local meteorological records from 2011 to 2018, the main winds direction is northeast and north (Fig. 2). This area is dominated with corn and the sample plot is a typical Gobi beach with coarse soil particles and natural vegetation in the sandy desert region is dominated by the psammophytes, such as *Nitraria tangutorum*, *Artemisia ordosica*, *Zygophyllum xanthoxylom (Bge.) Maxim*., *Ammopip-tanthus mongolicus (Cheng f.)*, *Haloxylon ammodendron (C.A.Mey.) Bge*., *Psammochloa villosa (Trin.) Bor*., *Phragmites aust-ralis Trin*., *Bassia dasyphylla Fisch. Et Mey*, *Suaeda glauca (Bge.)*, *Salsolacollina Pall*., *Agriophyllum squrrosum (L.) Moq*., *Holoxylon ammodendron*, *Kalidium foliatum*, *Reaumuria soongorica*, *Tamarix chinensis*, *Agropyron mongolicum*, *Glycyrrhiza uralensis*, *Sophora alopecuroides*, *Caragana korshinskii*., etc.

## Experiment Design

**Figure 3:**
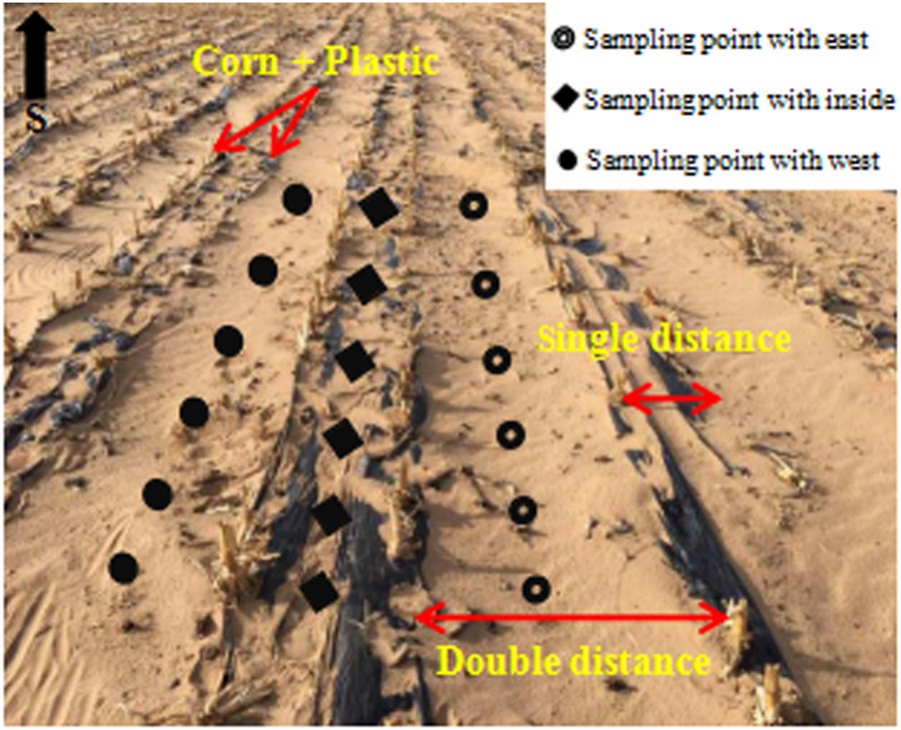
Test plot diagram of “double-row plastic film mulching in large ridges of maize”.

Before the sample plot was arranged, the sand dunes were flattened by bulldozer, and the leveling area was 2700 m^2^. Using the technique of “double-row plastic film mulching in large ridges of maize” (Fig. 3), single row of corn distance (single distance) was 10 cm, double row of corn distance (double distance) was 45 cm, plant distance was 5 cm, planting density of 3,809 trees/m^2^. At the same time, the black film with width of 85 cm and thickness of 0.05 mm was covered in order to protect corn from wind erosion and maintain soil water. When sowing, the organic fertilizer fermented with cow dung and straw at a high temperature of 90 °C was used as the bottom fertilizer. After the corn harvest in autumn, the height of corn stubble was 15 cm, and the black film was still covered. Corn was sown in early May 2017 and harvested at the end of September of that year.

5–11 April 2019, sampling was carried out on the east side, west side and double band (inside) of the compound sand barrier. Six samples were respectively selected in parallel at each location and all samples were taken from soil surface with 0–3 cm (Fig. 3). Three replicates were applied to each sample. At the same time, the control sample (CK) was collected on the bare sandy land. All samples was mixed together and placed in a sealed bag for later analysis. 63 sediment samples in total. Using the diagonal sampling method, 180 g soil samples was packed into plastic sealing bag for next test in laboratory. Research on the effect of the compound sand barrier on the surface wind erosion had of great practical significance because the compound sand barrier has been subjected to a plurality of wind seasons.

## Measurement of Sediment Grain-Size

Air drying, sieve off impurities and desalt of sediment samples were carried out in the Key Open Laboratory of National Forestry Administration for the Protection and Cultivation of Biological Resources in Sandy Land of Inner Mongolia Agricultural University^35^. Firstly, the dried samples were screened with a 2 mm soil sieve to the vegetation litter and the surface debris. 5 g soil sample was fully heated by adding 10 ml Hydrogen Peroxide and 10 ml Hydrochloric Acid and distilled water were added to completely remove carbonate from soil sample. After 24 hours, pH was tested repeatedly until the value was between 6.5 and 7.0. Finally, particle size was measured by using Mastersizer 3000 laser grain-size analyzer made by Malvern Company of the United Kingdom, combined with Hydro LV large capacity sample pool with relatively large sample size or wide particle size distribution^36^. The measurement range is 0.01–3500 μm and the accuracy is 0.6%. In addition, the repeatability is more than 0.5% and the reproducibility is higher than 1%. Each sample was repeatedly determined there times, and its arithmetic average was taken^1^.

The grain-size of the mechanical composition was divided into three grades according to the American (US-DA) standard, the clay (<0.005 mm), silt (0.005–0.063 mm) and sand (0.063–0.5 mm). Sand was further divided into: extremely fine sand (0.063–0.125 mm), fine sand (0.125–0.25 mm), medium sand (0.25–0.5 mm) and coarse sand (>0.5 mm)^23^.

## Calculation of the Grain-Size Parameter

According to the Udden-Wenworth grain-size standard and Kum-dein algorithm, the logarithmic transformation was carried out. The particles diameter (D, mm) corresponding to the cumulative volume fraction of sediment particles was converted into Φ value, which was convenient for drawing and calculation. The conversion formula is:

1 $$\Phi =-\,{\log }_{2}{\rm{D}}$$

Grain-size parameters (Average Grain Diameter (M_a_)_,_ Sorting Coefficient (σ), Skewness (SK) and Kurtosis (K_g_)) were calculated according the Folk-Ward method^24^.

## Calculation of The Average Distance of Sediment Grain-Size Accumulation Frequencies

Average distance of accumulation frequencies with sediment grain-size (d) can reflect the quality difference of the sediment and the cumulative frequency curve of the sediment grain-size, which can be used to support the determination of wind-erosion particle range. The calculation formula is as follows:

2 $$d=\sqrt{\sum {(P-\bar{P})}^{2}(K-1)}$$

Where d is the Average distance of accumulation frequencies with sediment grain-size; P is the accumulation frequency of sediment grain-size in a certain sample plot; $\overline{P}$ is the average accumulation frequency of sediments grain-size in different sample plot; K-1 is freedom degree and K = 4.

## Result and Analysis

### The surface grain-size distribution of sediment

The statistical results of the grain-size characteristics of sediment showed that the fine sand (44.87%) and medium sand (38.79%) were the main particles in the sand surface, especially the fine sand was the most. The silt was very low and not more than 2%, and there was not clay particle. The content of fine sand and very fine sand in the inner side of compound sand barrier was higher than that in the east side (77.70%) and the west side (78.86%), and the silt content was more than 0.26%. In general, the compound sand barrier was composed of the most fine sand, followed by extremely fine sand and medium sand, with the least coarse sand and silt particles. Obviously, due to the lack of effective protection, the mobile sandy land was in the direct contact layer between wind and sand, which made the fine sand in the surface layer eroded and the content of coarse sand and medium sand increased. However, because of the weakening of wind erosion caused by the blocking effect of the compound sand barrier, the loss of fine material was relatively less, and the content of coarse sand and medium sand was reduced accordingly (Table 1). In addition, in terms of grain-size characteristics, the existence of compound sand barrier can form a weak wind area and reduce the degree of wind erosion.

**Table 1:** The grain-size distribution with surface sediment of compound sand barrier.

Position	Clay (>8Φ)/%	Silt (4~8Φ)/%	Extremely fine sand (3~4Φ)/%	Fine sand (2~3Φ)/%	Medium sand (1~2Φ)/%	Coarse sand (<1Φ)/%
CK	0.00	0.17 ± 0.05^b^	5.57 ± 0.11^b^	44.87 ± 3.56^b^	38.79 ± 4.22^b^	10.60 ± 0.26^b^
East	0.00	0.20 ± 0.04^a^	25.36 ± 0.65^a^	52.34 ± 3.78^a^	17.69 ± 1.67^a^	4.41 ± 0.09^a^
Inside	0.00	0.25 ± 0.03^c^	19.63 ± 0.74^c^	61.56 ± 3.94^c^	16.54 ± 0.56^c^	2.02 ± 0.05^c^
West	0.00	0.26 ± 0.03^c^	27.52 ± 0.81^d^	51.34 ± 3.56^d^	18.95 ± 1.94^d^	1.93 ± 0.10^d^

The data in the table were Mean ± Standard Deviation. Small letters indicated that the particle content has significant differences at different location of compound sand barrier (P < 0.05).

### The grain-size parameters of sediment

**Figure 4:**
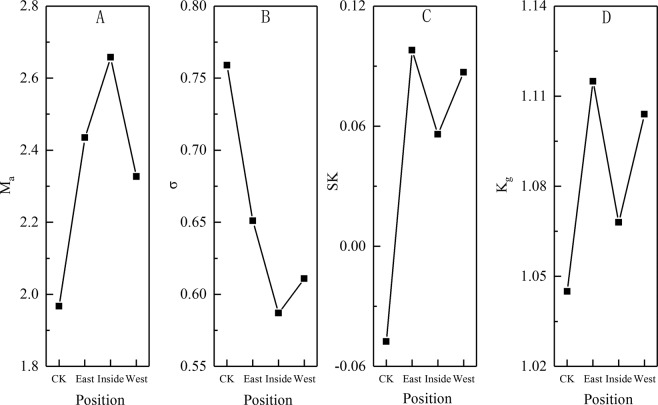
The grain-size parameters of surface aeolian sediment in compound sand barrier.

The Average Grain Diameter of the control surface sediment was 1.967Φ, which belonged to the medium sand. The east and west sides of compound sand barrier were 2.435Φ and 2.327Φ respectively, belonging to the fine sand; however, the Average Grain Diameter of the inner side of compound sand barrier was 2.658Φ, which belonged to the extremely fine sand (Fig. 4A). According to the division standard of Folk-Ward graphic method, the Sorting Coefficient was 0.587–0.759Φ, among which the sorting property of the control was the worst. With the setting of compound sand barrier, the Sorting Coefficient became better gradually, and the Sorting Coefficient in the inside was the best (Fig. 4B). In addition, the Skewness of the control was −0.048, which was the smallest. With the fixation of mobile sandy land, the Skewness in the east side of compound sand barrier was the largest with 0.098, which belonged to the positive Skewness, and the Skewness in the west and inner sides of compound sand barrier was 0.087 and 0.056, respectively, which belonged to the positive Skewness (Fig. 4C). The Kurtosis was 1.045–0.115, and the Kurtosis of frequency distribution curve was poor. The Kurtosis of the control was the smallest, the inner side of compound sand barrier was the second, and the west side and the east side were the last, both of which belonged to the leptokurtosis distribution (Fig. 4D).

### The frequency distribution curve of sediment

**Figure 5:**
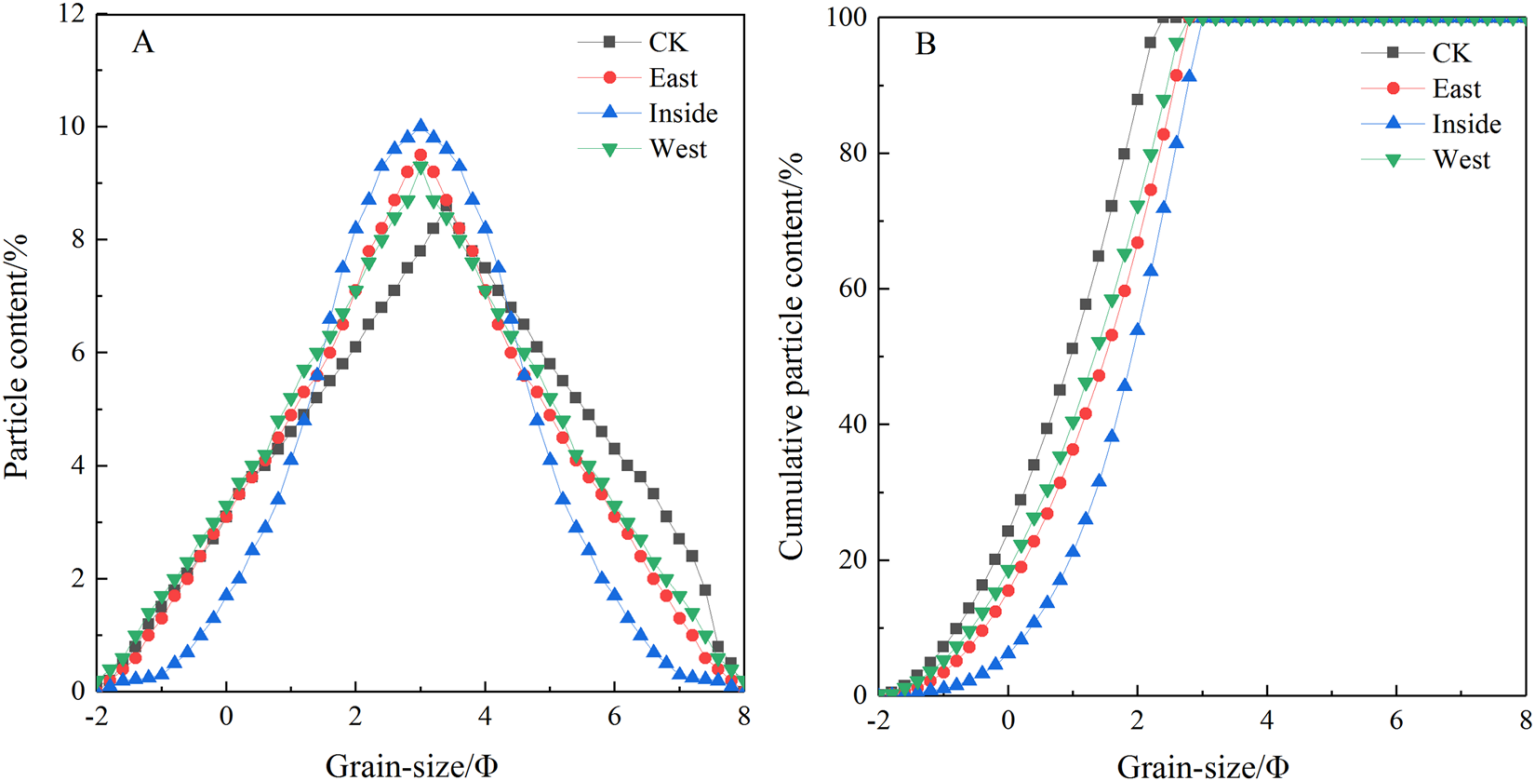
Frequency distribution curve of surface aeolian sediment in compound sand barrier.

The aeolian sediment at different location of the control and compound sand barrier had an unimodal type, and the Kurtosis developed in the positive and offset direction, and the overall Sorting Coefficient of the particles was better. Compared with the control, after laying the compound sand barrier, the distribution range of particles became steeper, the peak value improved and appeared ahead of time, and the composition of surface particles tended to one side of fine sand. The frequency distribution curve in the inner side of compound sand barrier was the steepest, and the proportion with fine sand of the surface layer decreased gradually from 10Φ to 9.5Φ and 9.3Φ respectively (Fig. 5A). At the same time, the cumulative frequency distribution curves on the east and west sides of the compound sand barrier were similar, which were gentler than those of the control. In addition, the cumulative frequency distribution curve on the control slowed down and delayed arrival the top of the curve. Moreover, the cumulative frequency distribution curve of the inside became steeper, the slope increased and reached the top of the curve ahead of time (Fig. 5B).

### The effect of wind environment and vegetation coverage on the surface sediments

**Figure 6:**
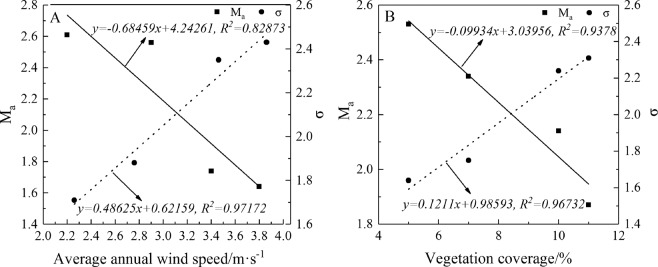
Relationship of among Average Grain Diameter (M_a_), average annual wind speed and vegetation coverage.

The spatial difference of surface sediments in sand area depends on the regional difference of wind environment and vegetation coverage^25^. The annual average wind velocity was respectively fitted linearly with the Sorting Coefficient and Average Grain Diameter. It showed that the average annual wind speed and the Average Grain Diameter had a negative correlation, while the average annual wind speed was positively correlated with the Sorting Coefficient. There was a high correlation among the average annual wind speed, the Sorting Coefficient and Average Grain Diameter, which were R^2^ = 0.82873 and R^2^ = 0.97172, respectively (Fig. 6A). Moreover, the correlation among vegetation coverage, the Sorting Coefficient and the Average Grain Diameter was higher, with R^2^ = 0.9378 and R^2^ = 0.96732, respectively. Vegetation coverage was negatively correlated with the Average Grain Diameter, while the Sorting Coefficient was positively correlated (Fig. 6B).

## Discussion and Conclusion

The grain-size characteristics of soil surface sediments under the corn straw and black film were analyzed. In this study we found that compound sand barrier was mainly composed of extremely fine sand and fine sand. And fine sand and extremely fine sand in the inner side were higher than the east and west sides of the compound sand barrier. Moreover, compound sand barrier could trap the moving sand and stabilize the original surface. Previous research showed that straw checkerboard could change coarse soil texture to fine because of increased the silt and clay content^1^. However, the results showed that the mobile sandy land was in the direct contact layer between wind and sand due to the lack of effective protection, which made fine sand in the surface eroded and the content of coarse and medium sand increased. It was consistent with the results reported by Buckley *et al*.^37^, which mobile sand dunes have a low silt and clay content and consequently constitute harsh ecology environments that are lower in available nutrients for plant growth^38–41^. Therefore, it is necessary and important to develop effective measures to prevent or abate wind erosion. Previous results have shown that sand barriers significantly increase the roughness of the underlying surface, reduce the wind speed and transport intensity, and form a stable and concave barrier^42–49^. Straw sand barriers increase the surface roughness, reduce the wind velocity, and gradually improve the soil moisture and promote the natural revegetation^50^. In addition, wind tunnel experiments proved that sand barriers could significantly decrease the wind velocity^51,52^. Also, our finding indicated that the existence of compound sand barrier could make it form a weak wind area and reduce wind erosion. Moreover, the effect of wind environment and vegetation coverage on the surface sediments showed that the average annual wind velocity and vegetation coverage was negatively correlated with the average grain-size, but positively correlated with the Sorting Coefficient. There was a high correlation among the annual wind speed, vegetation coverage, average grain-size and Sorting Coefficient, which indicated that the change of vegetation coverage and wind environment was the key factor leading to the difference of surface sediments in this area.

Different soils have varying erodibility due to differences of mechanical structure^53^. The method of particle-size distribution comparison (PSDC), proposed by Dong and Chen^54^, was used to estimate the wind erosion induced soil loss by comparing the change of the relative contents of erodible and non-erodible particles between the soil surface of farmland reclaimed from grassland and that of adjacent grassland. Dong and Li^55^ found that fine sand and medium sand with 1.3219Φ to 3.7370Φ were easily eroded particles, and coarse sand with 0.5146Φ to 1.3219Φ were relatively difficult to erode particles through the wind tunnel simulation. In this study, according to the Folk-Ward standard, the Sorting Coefficient was 0.587–0.759Φ. Due to the blocking effect of sand barriers, the average particle size was dominated with fine sand and the Sorting Coefficient became better gradually^56–59^. In addition, the Skewness belonged to the positive deviation and the Kurtosis presented leptokurtosis distribution. The range of wind erosion particles obtained in this study was basically consistent with that above. Further, the frequency distribution curve in the inside was the steepest, while the proportion of the fine sand of the surface layer in the east side and the west side of the compound sand barrier was gradually reduced. The frequency distribution curve of mobile sandy land gradually widened, the surface particles tended to coarse sand. And the cumulative frequency distribution curve of the inside became steeper, the slope increased and reached the top of the curve ahead of time. It is consistent with that previous studies have shown sediments in Ulan Buh Desert are mainly composed of coarser sands whose median grain size is greater than 0.08 mm.
