# Author Correction: Effects of Compound Sand Barrier for Habitat Restoration on Sediment Grain-size Distribution in Ulan Buh Desert

**DOI:** 10.1038/s41598-020-67065-8

**Published:** 2020-06-17

**Authors:** Xia Pan, Zhenyi Wang, Yong Gao

**Affiliations:** 1 College of Desert Control Science and Engineering, Inner Mongolia Agricultural University, Hohhot, 010018 China; 2 Wind Erosion Key Laboratory of Central and Government, Hohhot, 010018 China

Correction to: *Scientific Reports* 10.1038/s41598-020-59538-7, published online 13 February 2020

This Article contains a typographical error in the Site Description section.

“According to local meteorological records from 2011 to 2018, the main winds direction is northeast and north (Fig. 2).”

should read:

“According to local meteorological records from 2013 to 2017, the main winds direction is easterly and westerly (Fig. 2).”

Additionally, this Article contains errors in Figure 2. In Figure 2, the ranges of wind speed (m.s^−1^) are incorrect. The correct Figure 2 appears below as Figure 1.

**Figure 1:**
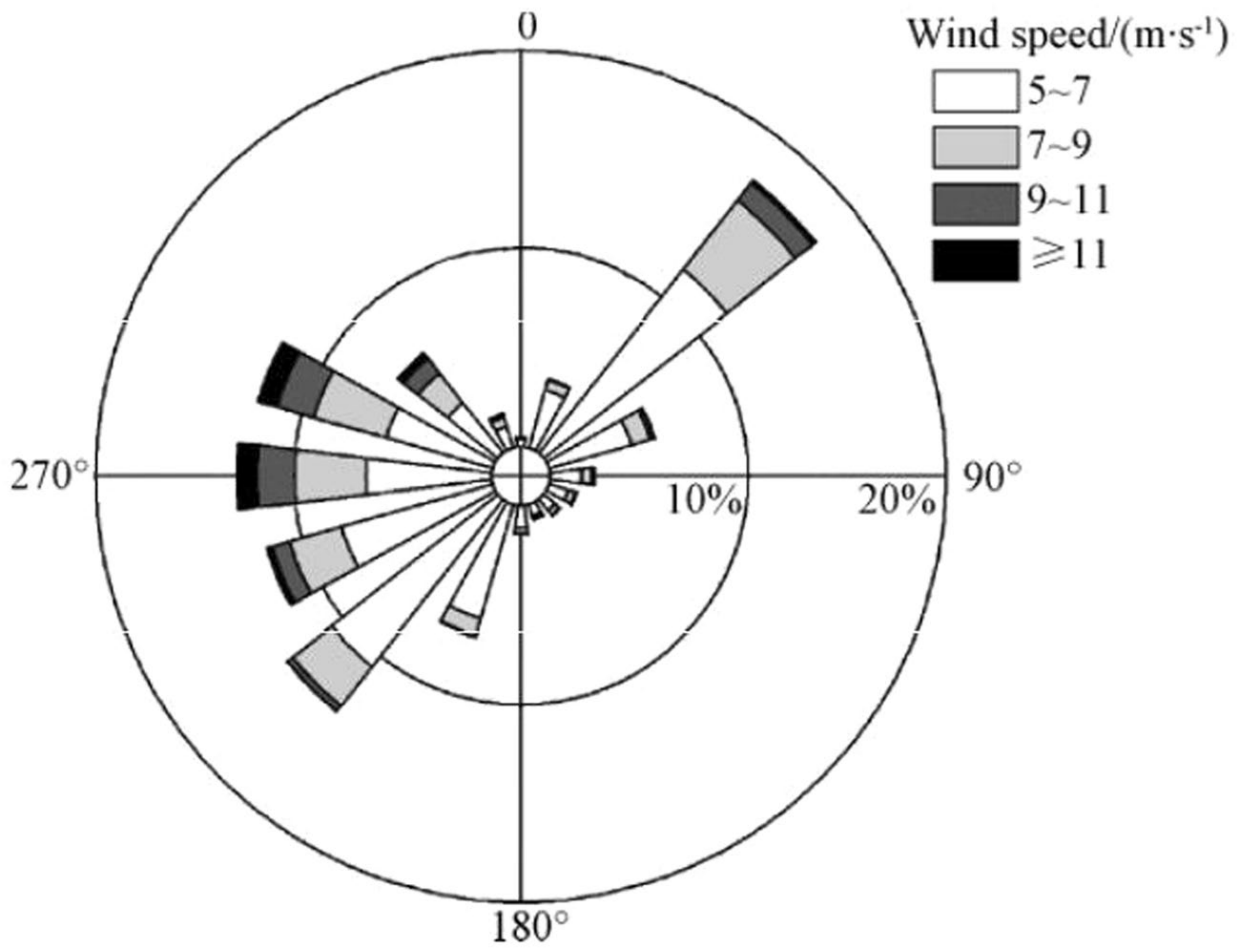
.

As a result, the Figure legend,

“Wind rose at northeastern margin region of Ulan Buh Desert from 2011 to 2018.”

should read:

“Wind rose at northeastern margin region of Ulan Buh Desert from 2013 to 2017”

